# Association of continuous glucose monitoring with in-hospital adverse outcomes among critically ill patients: a real-world propensity score–matched study

**DOI:** 10.3389/fendo.2026.1921773

**Published:** 2026-08-31

**Authors:** Zeyu Jiang, Yingying Zhang, Zhenhui Dong, Chong Wang, Jun Wang

**Affiliations:** 1 Department of Cardiology, The Affiliated Hospital of Qingdao University, Qingdao, China; 2 Intensive Care Unit, The Affiliated Hospital of Qingdao University, Qingdao, China; 3 Department of Cardiology, Dandong Central Hospital of China Medical University, Dandong, Liaoning, China; 4 Department of Emergency Medicine, The Affiliated Hospital of Qingdao University, Qingdao, China

**Keywords:** continuous glucose monitoring, glycemic variability, hypoglycemia, in-hospital mortality, intensive care unit, length of stay

## Abstract

**Background:**

Critically ill patients often experience substantial glycemic fluctuations, which are associated with adverse clinical outcomes. Conventional point-of-care glucose monitoring provides only intermittent measurements and may delay timely therapeutic adjustment. Continuous glucose monitoring (CGM) enables real-time assessment of glucose trends and may improve glycemic management in intensive care settings.

**Methods:**

This single-center, retrospective, real-world propensity score–matched cohort study included 592 critically ill adults admitted to the intensive care unit. Patients receiving CGM-guided glucose management were matched 1:1 with patients receiving standard point-of-care glucose monitoring. The primary outcome was in-hospital mortality. Secondary outcomes included ICU length of stay, hypoglycemia, severe hypoglycemia, mechanical ventilation duration, and nosocomial infection. Outcomes were analyzed using regression models appropriate to outcome type, with adjustment for prespecified covariates.

**Results:**

After propensity score matching, 296 patients were included in the CGM group and 296 in the control group. Compared with standard point-of-care monitoring, CGM use was associated with lower in-hospital mortality (9.5% vs 15.5%; adjusted odds ratio [aOR] = 0.61, 95% CI 0.38–0.98; P = 0.027) and fewer severe hypoglycemic events (4.7% vs 10.8%; aOR = 0.46, 95% CI 0.25–0.85; P = 0.009). The CGM group also had a shorter median ICU length of stay (7 days [IQR 5–10] vs 9 days [IQR 6–13]; P = 0.021) and a lower incidence of nosocomial infection (12.2% vs 17.9%; aOR = 0.63, 95% CI 0.40–0.99; P = 0.048).

**Conclusion:**

In this retrospective propensity score–matched cohort study, CGM-guided glucose management was associated with improved glycemic safety and more favorable in-hospital outcomes among critically ill adults who remained in the ICU for at least 72 hours. Because of the observational design, survivor-selection bias, residual confounding, and differences in measurement density, these findings should not be interpreted as evidence of causal benefit. Prospective multicenter studies are needed.

## Introduction

Diabetes has emerged as a major global health challenge, imposing substantial social and economic burdens (1–3). Its prevalence continues to rise in parallel with shifts in lifestyle and dietary patterns. Achieving and maintaining optimal glycemic control remains central to diabetes management, as it prevents or delays the development of complications (4). CGM has gained increasing attention as a potential tool for inpatient glucose management, although questions regarding its implementation and clinical readiness remain (5). However, a large proportion of individuals, particularly those requiring insulin, fail to meet their glycemic targets in real-world clinical practice (6).

Glycemic control represents an even greater challenge in critically ill patients (7). Both hyperglycemia and hypoglycemia, as well as excessive glycemic variability, are strongly associated with poor outcomes, including increased mortality (8). Intermittent point-of-care glucose testing, the current standard in most intensive care units (ICU), provides only sporadic measurements that may overlook clinically significant fluctuations and delay therapeutic responses (9). This monitoring gap can result in suboptimal metabolic control and contribute to prolonged ICU stays, higher rates of nosocomial infection, and increased mortality (10).

Continuous glucose monitoring (CGM) technologies, either intermittent-scanning (isCGM) or real-time (rtCGM) systems, allow minimally invasive, continuous tracking of interstitial glucose levels without the need for repeated finger-stick testing (11). These systems overcome key limitations of conventional monitoring and enable more timely interventions. Although CGM has demonstrated clear benefits in outpatient diabetes management, its clinical value and safety profile in critically ill populations remain uncertain (12). Given the complex and high-risk nature of the ICU environment, rigorous evaluation is essential. Therefore, this retrospective propensity score–matched cohort study was designed to evaluate whether CGM-guided glucose management was associated with improved in-hospital outcomes among critically ill adults.

## Methods

### Study design and population

This was a single-center, retrospective, real-world, propensity score–matched cohort study conducted in the intensive care unit of The Affiliated Hospital of Qingdao University. Data were retrieved from the electronic medical record system between January 1, 2021, and December 31, 2023. Adult critically ill patients were eligible if they were aged 18–80 years and had an ICU stay of at least 72 hours.

**Figure 1:**
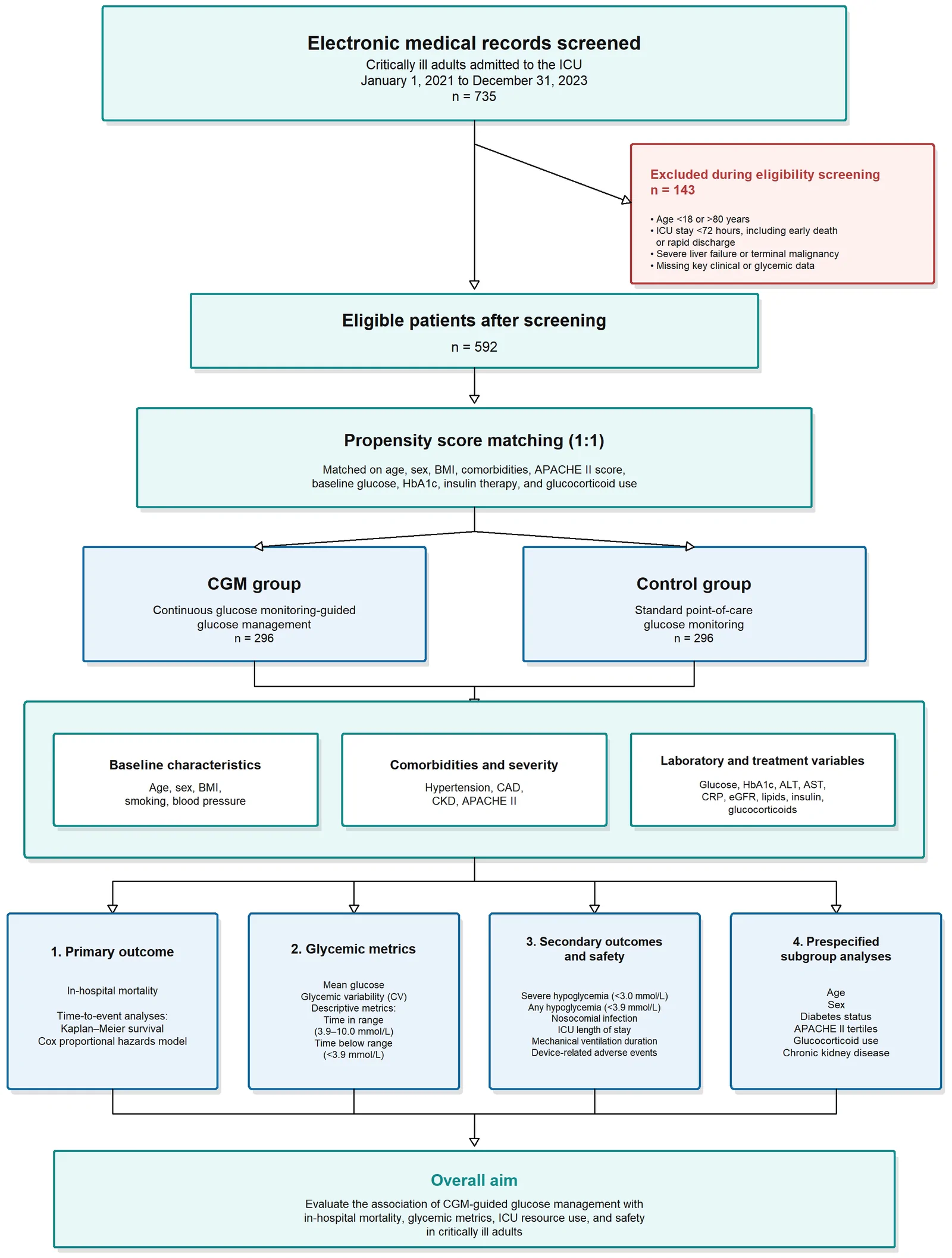
Study flow and analytical framework.

Patients were classified according to whether they received continuous glucose monitoring-guided glucose management or standard point-of-care glucose monitoring during ICU hospitalization. Patients were excluded if they had an ICU stay shorter than 72 hours, severe liver failure, terminal malignancy, or missing key clinical or glycemic information. The 72-hour criterion was prespecified to permit sufficient monitoring but necessarily excluded early deaths and rapid discharges. Of the 735 patients screened, 143 were excluded during eligibility assessment. The remaining patients were matched 1:1, resulting in a final propensity score–matched cohort of 592 patients, with 296 patients in each group (**Figure 1**).

### Ethical considerations

This study was conducted in accordance with the principles of the Declaration of Helsinki. The study protocol was reviewed and approved by the Ethics Committee of The Affiliated Hospital of Qingdao University (Approval No. QYFY WZLL 30709). Patient confidentiality was strictly maintained throughout the study, and all personal identifiers were removed from the analytic dataset.

The requirement for written informed consent was waived because of the retrospective nature of the study and the use of deidentified data.

### Data extraction and outcome

Baseline demographic and clinical characteristics were extracted from the electronic medical record system and the ICU clinical database. Extracted variables included age, sex, body mass index, duration of diabetes, smoking status, comorbidities, APACHE II score, vital signs, laboratory parameters, insulin therapy, glucocorticoid use, and cardiometabolic risk factors.

The primary outcome was in-hospital mortality. Secondary clinical outcomes were ICU length of stay, mechanical ventilation duration, any hypoglycemia, severe hypoglycemia, and nosocomial infection; device-related adverse events were safety outcomes. Any hypoglycemia was defined as glucose <3.9 mmol/L and severe hypoglycemia as glucose <3.0 mmol/L. Nosocomial infection was an infection occurring at least 48 hours after ICU admission and included ventilator-associated pneumonia (VAP), bloodstream infection (BSI), and catheter-associated urinary tract infection (CAUTI), identified under institutional surveillance procedures based on CDC/NHSN criteria (13).

VAP required a new or progressive pulmonary infiltrate at least 48 hours after initiation of mechanical ventilation, at least two compatible clinical findings (temperature abnormality, leukocytosis/leukopenia, or purulent respiratory secretions), and supporting lower-respiratory-tract microbiology. BSI required a recognized pathogen from at least one blood culture unrelated to another infection site, or a common skin commensal from at least two separately drawn cultures with compatible clinical findings and no other source. Central-line association required a central venous catheter for more than 48 hours before onset or removal within the preceding 48 hours.

CAUTI required an indwelling urinary catheter for more than 2 days at the event, compatible urinary or systemic symptoms, and a urine culture of at least 10^5 CFU/mL with no more than two identified species. All infection diagnoses were confirmed by treating physicians.

Secondary glycemic-management measures included mean glucose and glycemic variability, assessed using the coefficient of variation (CV). For CGM patients, time in range (TIR) was defined as the percentage of monitored time between 3.9 and 10.0 mmol/L and time below range (TBR) as the percentage of monitored time below 3.9 mmol/L. In the POCT group, only the proportions of discrete measurements within and below these ranges could be calculated; these proportions are not equivalent to CGM-derived TIR or TBR and were treated as exploratory descriptive measures.

The institutional therapeutic glucose target was generally 8.0–10.0 mmol/L and was individualized according to baseline glycemic status, nutritional support, insulin requirements, glucocorticoid exposure, clinical condition, and hypoglycemia risk. The analytic range of 3.9–10.0 mmol/L was used to summarize glucose exposure and was not a mandatory therapeutic target. Glucose data were collected from initiation of the assigned monitoring modality until ICU discharge, death, discontinuation of monitoring, or the end of the available observation period, whichever occurred first.

### Continuous glucose monitoring protocol

Patients in the CGM group underwent glucose monitoring using the FreeStyle Libre H Flash Glucose Monitoring System (Abbott Diabetes Care Ltd., Witney, Oxon, United Kingdom), a hospital-use intermittently scanned continuous glucose monitoring system. The sensor was applied to the posterior upper arm by trained ICU nurses in accordance with the manufacturer’s instructions and could be worn for up to 14 days. When continued monitoring was required beyond the recommended wear period, the sensor was replaced.

The system measured interstitial glucose concentrations and generated longitudinal glucose profiles throughout the monitoring period. Glucose data were accessed using the hospital reader and were reviewed by the treating clinical team in conjunction with point-of-care blood glucose measurements. Insulin therapy was adjusted according to the institutional ICU glycemic-management protocol, incorporating glucose values, glucose trends, nutritional intake, insulin administration, and the patient’s risk of hypoglycemia.

CGM readings were not used as the sole basis for treatment decisions when they were inconsistent with the patient’s symptoms or clinical condition. In patients with shock, severe tissue hypoperfusion, substantial edema, rapidly changing glucose concentrations, suspected sensor malfunction, or high-dose vasopressor therapy, confirmatory point-of-care or laboratory plasma glucose testing was performed before treatment modification.

In the POCT group, glucose was routinely measured every 4 hours, with additional measurements after insulin adjustment, when hypoglycemia was suspected, or when glucose was changing rapidly. Mean glucose was the arithmetic mean of all available POCT measurements during the observation period. CV was calculated as the standard deviation divided by the mean and multiplied by 100%.

### Clinical allocation of glucose monitoring modality

During the study period, the choice of CGM or standard POCT was determined by the treating ICU physicians according to routine clinical practice rather than by random assignment. CGM was generally considered for patients requiring intensive glucose monitoring because of persistent hyperglycemia, marked glycemic variability, insulin infusion therapy, or an anticipated need for frequent glucose assessment. The final decision was based on the treating physician’s clinical judgment together with device availability. No formal institutional protocol mandated the use of CGM for any specific patient subgroup.

### Propensity score matching and statistical analysis

Propensity scores were estimated using a logistic regression model including clinically relevant baseline variables: age, sex, body mass index, hypertension, coronary artery disease, chronic kidney disease, APACHE II score, baseline glucose, HbA1c, insulin therapy, and glucocorticoid use. Patients in the CGM and control groups were matched in a 1:1 ratio using nearest-neighbor matching without replacement. Covariate balance before and after matching was assessed using standardized mean differences, with a standardized mean difference <0.10 considered acceptable balance.

All statistical tests were two-sided, and a P value <0.05 was considered statistically significant. Continuous variables are presented as mean ± standard deviation or median with interquartile range, as appropriate. Categorical variables are presented as numbers and percentages. Baseline characteristics were compared between groups using t tests or Wilcoxon rank-sum tests for continuous variables and χ² tests or Fisher’s exact tests for categorical variables.

For binary outcomes, including in-hospital mortality, severe hypoglycemia, any hypoglycemia, and nosocomial infection, adjusted odds ratios and 95% confidence intervals were estimated using generalized estimating equation models with a binomial distribution and logit link. For count outcomes, including ICU length of stay and mechanical ventilation duration, negative binomial regression models were used to estimate adjusted incidence rate ratios and 95% confidence intervals. Time-to-event outcomes were analyzed using Kaplan–Meier curves, log-rank tests, and Cox proportional hazards models to estimate adjusted hazard ratios and 95% confidence intervals. The proportional hazards assumption was assessed using Schoenfeld residuals.

Sensitivity analyses were performed with additional adjustment for APACHE II score, chronic kidney disease, and glucocorticoid use, where these variables were not already included in the primary outcome-specific models. The matched-pair structure was accounted for using patient-pair clustering and cluster-robust standard errors, as appropriate.

Patient-level mean glucose and CV were analyzed as secondary glycemic-management measures using multivariable linear regression adjusted for age and sex. Because CGM provides continuous data whereas POCT provides discrete measurements, CGM-derived TIR and TBR were not considered directly comparable with the corresponding POCT proportions. Any descriptive comparison of these measures was considered exploratory and was not interpreted as a comparative treatment effect. Analyses were performed using complete-case data in R version 4.3.3.

## Results

### Baseline characteristics of the study population

After propensity score matching, a total of 592 critically ill patients were included in the final analytical cohort, with 296 patients in the CGM group and 296 patients in the control group. Baseline demographic and clinical characteristics were well balanced between the two groups (**Table 1**).

**Table 1:** Baseline characteristics of the study population.

Variable	CGM group (n = 296)	Control group (n = 296)	P value
Age, years (mean ± SD)	63.8 ± 11.2	64.1 ± 10.9	0.76
Male, n (%)	175 (59.1)	179 (60.5)	0.72
Body mass index, kg/m² (mean ± SD)	24.7 ± 3.9	24.5 ± 4.2	0.58
Duration of diabetes, years (median, IQR)	8 (5–12)	8 (5–11)	0.64
Current smoker, n (%)	90 (30.4)	94 (31.8)	0.73
Hypertension, n (%)	203 (68.6)	208 (70.3)	0.67
Coronary artery disease, n (%)	84 (28.4)	89 (30.1)	0.65
Chronic kidney disease, n (%)	57 (19.3)	61 (20.6)	0.74
APACHE II score (mean ± SD)	18.3 ± 5.4	18.0 ± 5.1	0.59
Heart rate, beats/min (mean ± SD)	88.4 ± 13.6	89.1 ± 14.0	0.62
Respiratory rate, breaths/min (mean ± SD)	21.8 ± 3.2	22.1 ± 3.4	0.47
ALT, U/L (mean ± SD)	32.5 ± 16.8	31.9 ± 17.2	0.73
AST, U/L (mean ± SD)	35.8 ± 18.9	36.2 ± 19.4	0.84
Uric acid, μmol/L (mean ± SD)	345.6 ± 88.2	351.7 ± 90.1	0.49
C-reactive protein, mg/L (median, IQR)	46.3 (29.5–82.1)	48.7 (30.2–85.9)	0.61
eGFR, mL/min/1.73 m² (mean ± SD)	78.9 ± 22.4	77.6 ± 21.9	0.58
Blood glucose, mmol/L (mean ± SD)	9.8 ± 3.2	9.7 ± 3.0	0.82
HbA1c, % (mean ± SD)	7.9 ± 1.4	7.8 ± 1.3	0.67
Glucocorticoid use, n (%)	74 (25.0)	78 (26.4)	0.71
Total cholesterol, mmol/L (mean ± SD)	4.45 ± 0.96	4.41 ± 0.92	0.68
Triglycerides, mmol/L (median, IQR)	1.47 (1.02–2.01)	1.52 (1.05–2.07)	0.57
LDL-C, mmol/L (mean ± SD)	2.41 ± 0.78	2.45 ± 0.81	0.66
HDL-C, mmol/L (mean ± SD)	1.12 ± 0.29	1.10 ± 0.31	0.49
Insulin therapy, n (%)	172 (58.1)	168 (56.8)	0.78
Blood pressure, mmHg (mean ± SD)	128 ± 18 / 76 ± 10	129 ± 17 / 77 ± 9	0.55
Serum creatinine, μmol/L (mean ± SD)	95.4 ± 36.8	96.1 ± 35.7	0.81

The mean age was 63.8 ± 11.2 years in the CGM group and 64.1 ± 10.9 years in the control group, and the proportion of male patients was similar between groups (59.1% vs 60.5%). Body mass index was also comparable between the CGM and control groups (24.7 ± 3.9 vs 24.5 ± 4.2 kg/m²). The median duration of diabetes was 8 years in both groups. There were no significant differences in smoking status or major comorbidities, including hypertension, coronary artery disease, and chronic kidney disease.

Disease severity at ICU admission was similar between the two groups, as reflected by comparable APACHE II scores (18.3 ± 5.4 vs 18.0 ± 5.1). Vital signs and laboratory parameters, including heart rate, respiratory rate, alanine aminotransferase, aspartate aminotransferase, uric acid, C-reactive protein, estimated glomerular filtration rate, blood glucose, HbA1c, lipid profiles, and serum creatinine, were also comparable. In addition, the proportions of patients receiving insulin therapy and glucocorticoid treatment did not differ significantly between groups. These findings indicate good baseline comparability between the CGM and control groups after matching.

### Major clinical outcome landscape associated with CGM use

**Figure 2:**
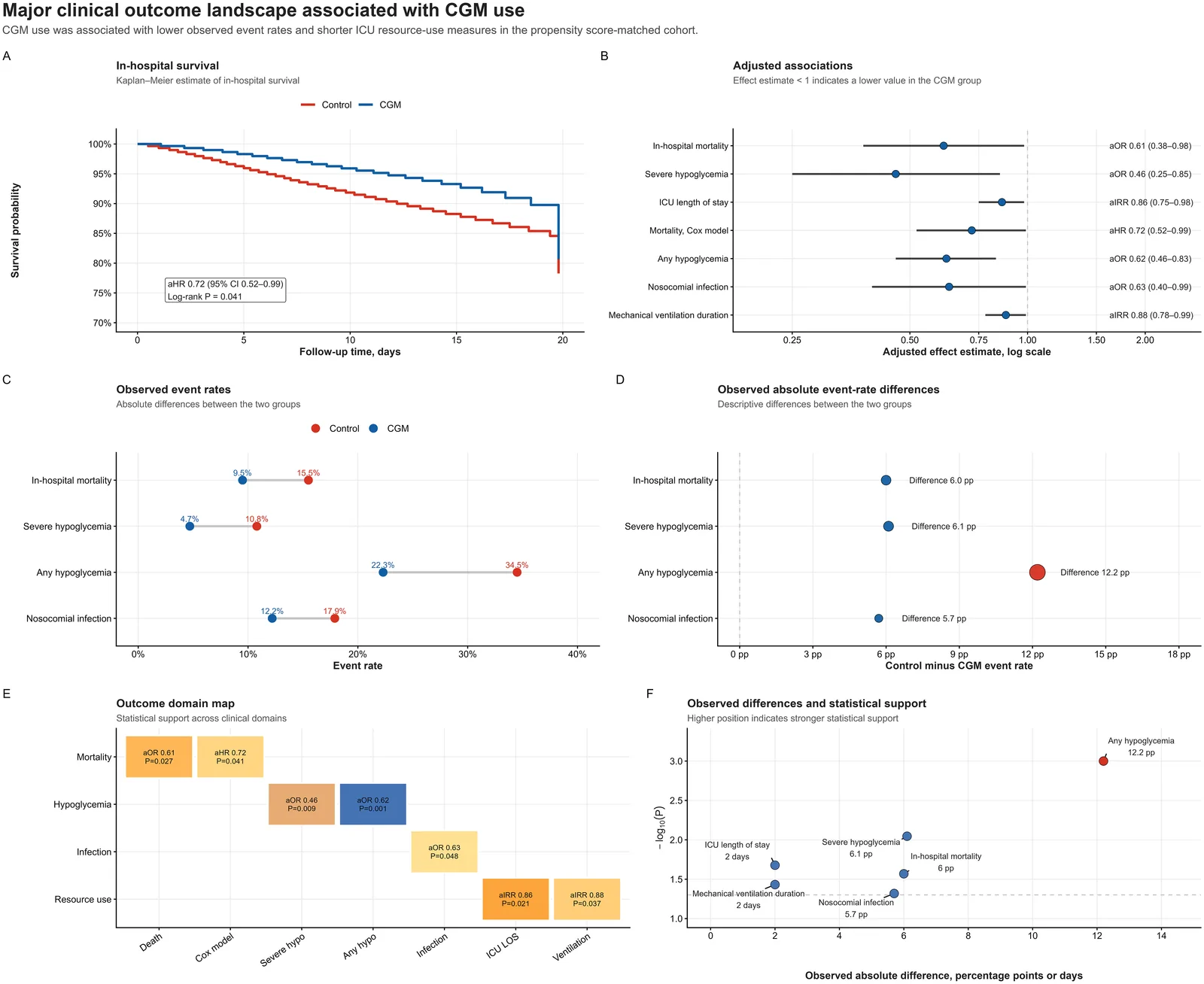
Major clinical outcomes associated with CGM use. **(A)** Kaplan–Meier curves comparing in-hospital survival between the CGM and control groups, with the adjusted hazard ratio from the Cox proportional hazards model. **(B)** Forest plot of adjusted associations for mortality, hypoglycemia, nosocomial infection, ICU length of stay, and mechanical ventilation duration. Estimates below 1 indicate lower odds, hazards, or incidence rates in the CGM group, according to the outcome-specific model. **(C)** Observed event rates in the two groups. **(D)** Descriptive absolute event-rate differences, calculated as the control-group rate minus the CGM-group rate. **(E)** Outcome-domain map summarizing adjusted estimates and statistical support across mortality, hypoglycemia, infection, and resource-use outcomes. **(F)** Observed absolute differences plotted against statistical support. CGM, continuous glucose monitoring; ICU, intensive care unit.

**Figure 2** and **Table 2** summarize the association between CGM use and major clinical outcomes. In the propensity score–matched cohort, patients in the CGM group showed a lower incidence of in-hospital mortality than those in the control group. In-hospital mortality occurred in 28 of 296 patients in the CGM group and 46 of 296 patients in the control group, corresponding to event rates of 9.5% and 15.5%, respectively. After adjustment for age and sex, CGM use was associated with lower odds of in-hospital mortality, with an adjusted odds ratio of 0.61 (95% CI, 0.38–0.98; P = 0.027).

**Table 2:** Major clinical outcomes.

Outcome	CGM (n = 296)	Control (n = 296)	Unadjusted effect	Adjusted effect	P value
In-hospital mortality, n (%)	28 (9.5)	46 (15.5)	RR 0.61 (0.39–0.95)	aOR 0.61 (0.38–0.98)*	0.027
Severe hypoglycemia <3.0 mmol/L, n (%)	14 (4.7)	32 (10.8)	RR 0.44 (0.24–0.80)	aOR 0.46 (0.25–0.85)*	0.009
ICU length of stay, days, median (IQR)	7 (5–10)	9 (6–13)	—	aIRR 0.86 (0.75–0.98)†	0.021
Kaplan–Meier survival (CGM vs POCT)	—	—	Log-rank χ² = 4.1	aHR 0.72 (0.52–0.99)‡	0.041

RR, risk ratio; aOR, adjusted odds ratio; aIRR, adjusted incidence rate ratio; aHR, adjusted hazard ratio; IQR, interquartile range.

*Multivariable GEE (binomial) adjusted for age and sex (primary model). † Negative binomial regression for length of stay, adjusted for age, sex, APACHE II, chronic kidney disease, glucocorticoid use.

‡Cox proportional hazards model adjusted for age and sex.

Severe hypoglycemia was also less frequent in the CGM group than in the control group. Severe hypoglycemic events occurred in 14 patients in the CGM group and 32 patients in the control group, with corresponding rates of 4.7% and 10.8%, respectively. In the adjusted model, CGM use was associated with lower odds of severe hypoglycemia (adjusted odds ratio, 0.46; 95% CI, 0.25–0.85; P = 0.009).

The CGM group also had a shorter ICU length of stay than the control group. The median ICU length of stay was 7 days (IQR, 5–10) in the CGM group and 9 days (IQR, 6–13) in the control group. Negative binomial regression showed that CGM use was associated with a shorter ICU stay, with an adjusted incidence rate ratio of 0.86 (95% CI, 0.75–0.98; P = 0.021).

Kaplan–Meier survival analysis further demonstrated higher in-hospital survival probability among patients in the CGM group compared with those in the control group. The log-rank test showed a significant between-group difference, and the Cox proportional hazards model indicated that CGM use was associated with lower in-hospital mortality risk, with an adjusted hazard ratio of 0.72 (95% CI, 0.52–0.99; P = 0.041).

### Secondary glycemic-management measures during the ICU stay

**Figure 3:**
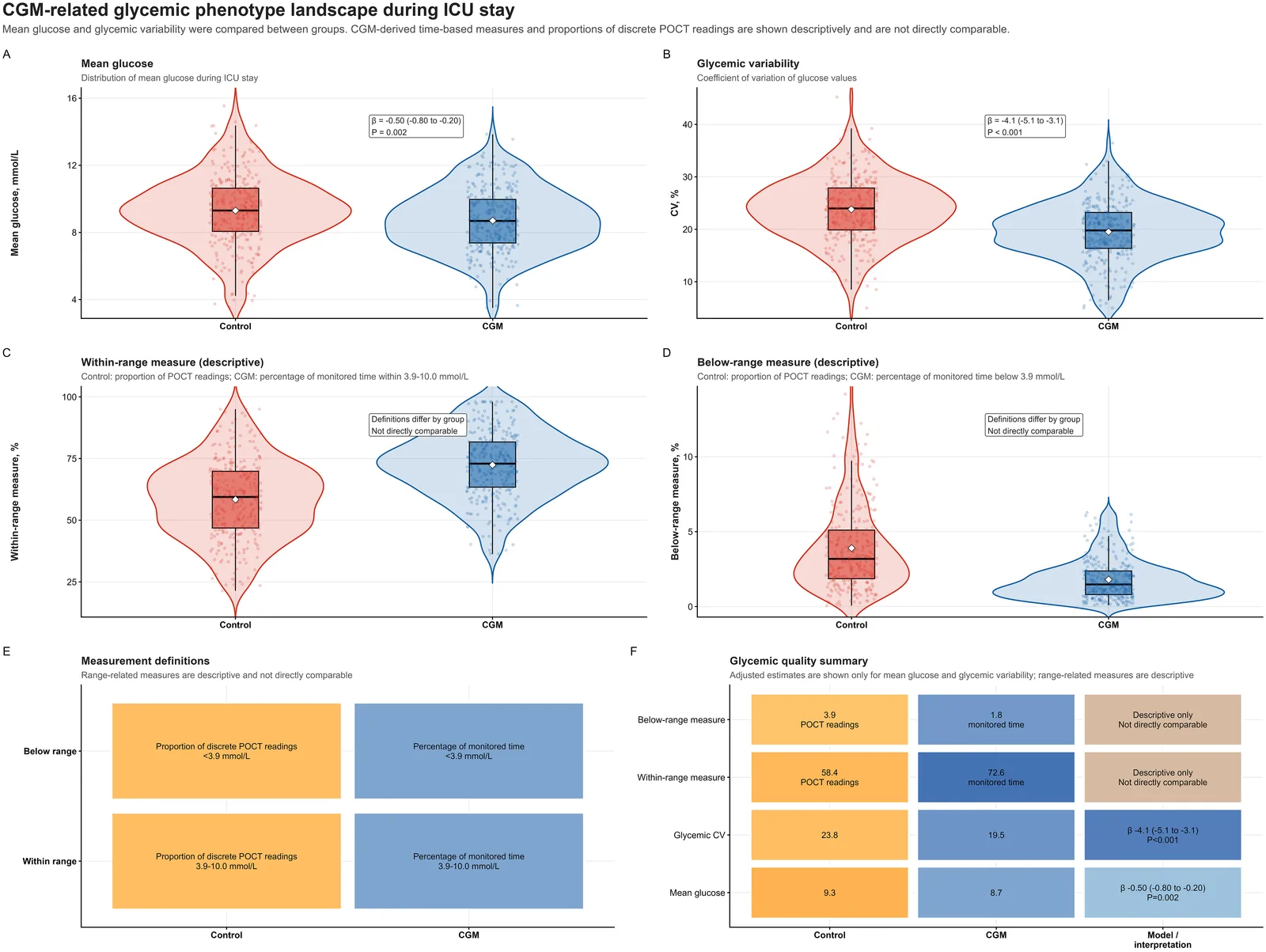
Glycemic measures during the ICU stay by monitoring group. **(A)** Distribution of patient-level mean glucose, with the adjusted between-group difference. **(B)** Distribution of glycemic variability, measured using the coefficient of variation, with the adjusted between-group difference. **(C)** Within-range measures shown descriptively as the proportion of discrete POCT readings in the control group and the percentage of monitored time in the CGM group. **(D)** Below-range measures shown descriptively using the corresponding group-specific definitions. **(E)** Definitions of the range-related measures used in the control and CGM groups. **(F)** Observed group values and adjusted estimates for mean glucose and glycemic variability; range-related measures are presented descriptively only. CGM-derived time-based measures and proportions of discrete POCT readings are not equivalent and should not be interpreted as directly comparable treatment effects. CGM, continuous glucose monitoring; CV, coefficient of variation; POCT, point-of-care testing.

**Figure 3** and **Table 3** summarize secondary glycemic-management measures. Mean glucose and CV are reported for both groups. CGM-derived TIR/TBR and the proportions of discrete POCT measurements within/below range are shown descriptively but are not treated as equivalent measures or as evidence of a comparative treatment effect.

**Table 3:** Secondary outcomes.

Outcome	CGM (n = 296)	Control (n = 296)	Unadjusted effect	Adjusted effect	P value
Mean glucose, mmol/L (mean ± SD)	8.7 ± 1.9	9.3 ± 2.1	Δ −0.6	β −0.50 (−0.80 to −0.20)§	0.002
Glycemic variability (CV, %), mean ± SD	19.5 ± 5.6	23.8 ± 6.1	Δ −4.3	β −4.1 (−5.1 to −3.1)§	<0.001
Time in range 3.9–10.0 mmol/L, %	CGM-derived 72.6	POCT measurement proportion 58.4	Not directly comparable	Exploratory descriptive comparison	—
Time below range <3.9 mmol/L, %	CGM-derived 1.8	POCT measurement proportion 3.9	Not directly comparable	Exploratory descriptive comparison	—
Any hypoglycemia <3.9 mmol/L, n (%)	66 (22.3)	102 (34.5)	RR 0.65 (0.50–0.84)	aOR 0.62 (0.46–0.83)*	0.001
Nosocomial infection, n (%)	36 (12.2)	53 (17.9)	RR 0.68 (0.46–0.99)	aOR 0.63 (0.40–0.99)*	0.048
Mechanical ventilation duration, days, median (IQR)	4 (3–7)	6 (4–9)	—	aIRR 0.88 (0.78–0.99)†	0.037
Device-related adverse events, n (%)	3 (1.0)	—	—	—	—

pp, percentage points; CV, coefficient of variation.

*Multivariable GEE (binomial) adjusted for age and sex.

†Negative binomial regression adjusted for age, sex, APACHE II, glucocorticoid use.

§Multivariable linear regression adjusted for age and sex.

CGM-derived TIR and TBR represent time-based continuous measures. The corresponding POCT values represent proportions of discrete measurements and are not equivalent; no causal or inferential between-group interpretation should be made.

Mean glucose was lower in the CGM group than in the control group (8.7 ± 1.9 vs 9.3 ± 2.1 mmol/L). In the multivariable linear regression model adjusted for age and sex, the adjusted mean difference was −0.50 mmol/L (95% CI, −0.80 to −0.20; P = 0.002). Glycemic variability, measured by the coefficient of variation, was also lower in the CGM group than in the control group (19.5% ± 5.6% vs 23.8% ± 6.1%), with an adjusted difference of −4.1 percentage points (95% CI, −5.1 to −3.1; P < 0.001).

CGM-derived TIR was 72.6%, whereas 58.4% of discrete POCT measurements were within 3.9–10.0 mmol/L. CGM-derived TBR was 1.8%, whereas 3.9% of POCT measurements were below 3.9 mmol/L. These quantities were generated by different measurement strategies and are therefore presented descriptively; they should not be interpreted as directly comparable estimates of time spent within or below range.

The range-related panels in **Figure 3** are descriptive and should be interpreted cautiously because CGM and POCT differ substantially in measurement density. The observed differences do not establish that changes in glucose exposure or variability mediated the association between monitoring strategy and clinical outcomes.

### Secondary clinical outcomes and safety

Secondary clinical outcomes are summarized in **Table 3**. Any hypoglycemia occurred less frequently in the CGM group than in the control group. A total of 66 patients in the CGM group and 102 patients in the control group experienced any hypoglycemia, corresponding to rates of 22.3% and 34.5%, respectively. After adjustment, CGM use was associated with lower odds of any hypoglycemia (adjusted odds ratio, 0.62; 95% CI, 0.46–0.83; P = 0.001).

Nosocomial infection was also less common among patients in the CGM group. Nosocomial infection occurred in 36 patients in the CGM group and 53 patients in the control group, with event rates of 12.2% and 17.9%, respectively. In the adjusted model, CGM use was associated with lower odds of nosocomial infection (adjusted odds ratio, 0.63; 95% CI, 0.40–0.99; P = 0.048).

Mechanical ventilation duration was shorter in the CGM group than in the control group. The median duration of mechanical ventilation was 4 days (IQR, 3–7) in the CGM group and 6 days (IQR, 4–9) in the control group. Negative binomial regression showed that CGM use was associated with a shorter duration of mechanical ventilation, with an adjusted incidence rate ratio of 0.88 (95% CI, 0.78–0.99; P = 0.037).

Regarding device safety, CGM-related adverse events were uncommon. Only 3 patients in the CGM group experienced device-related adverse events, accounting for 1.0% of CGM users. These consisted of mild local skin irritation at the sensor site (n=2) and premature sensor detachment requiring replacement (n=1). No serious device-related adverse events were observed.

### ICU resource use, safety, and subgroup robustness associated with CGM use

**Figure 4:**
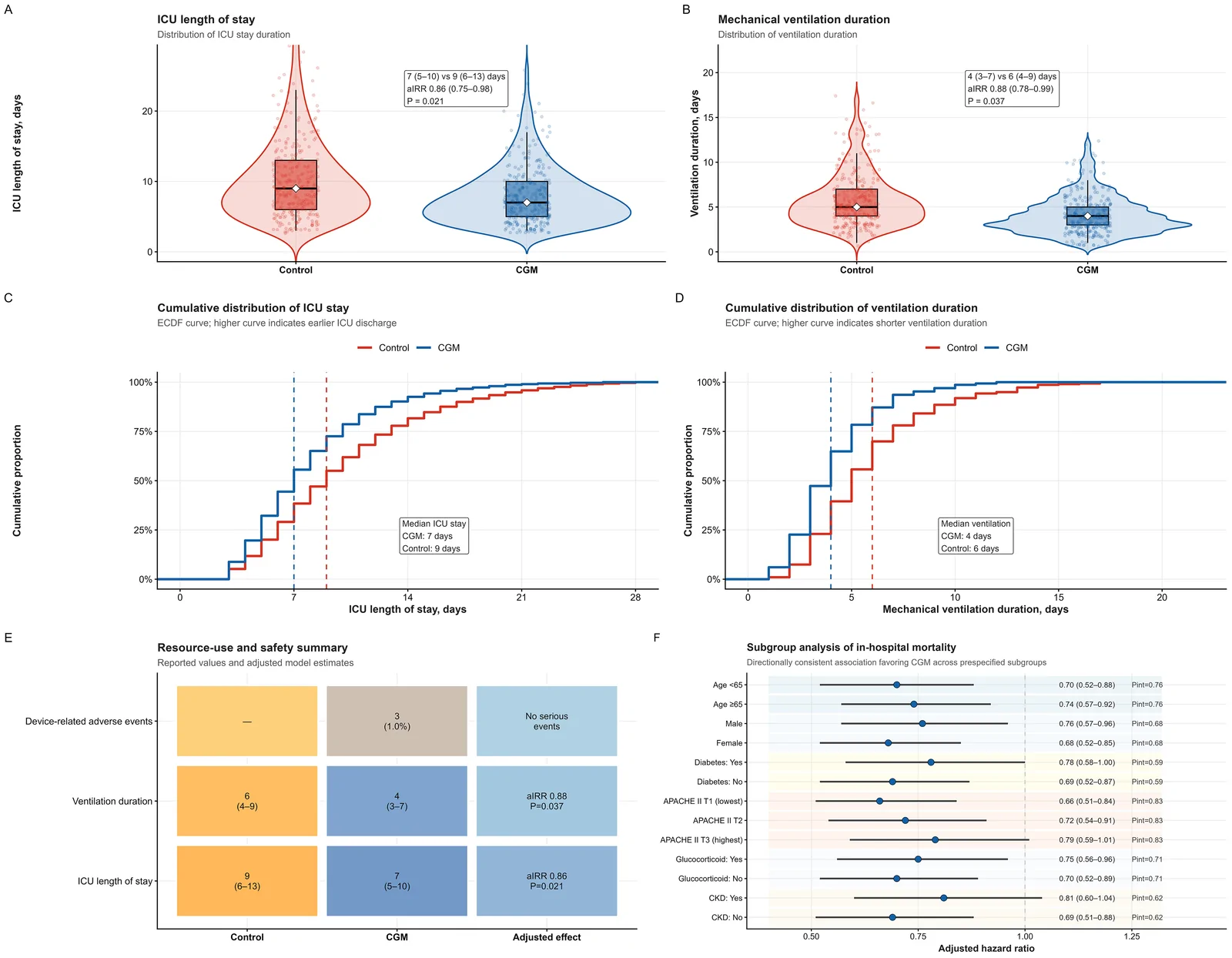
ICU resource use, safety, and subgroup analyses associated with CGM use. **(A)** Distributions of ICU length of stay in the control and CGM groups, with the adjusted incidence rate ratio. **(B)** Distributions of mechanical ventilation duration in the two groups, with the adjusted incidence rate ratio. **(C)** Empirical cumulative distribution curves for ICU length of stay. **(D)** Empirical cumulative distribution curves for mechanical ventilation duration. **(E)** Summary of observed ICU resource-use measures, adjusted estimates, and device-related adverse events. **(F)** Adjusted hazard ratios for in-hospital mortality across prespecified subgroups; interaction P values assess heterogeneity of the association. CGM, continuous glucose monitoring; ICU, intensive care unit.

**Figure 4** presents ICU resource utilization, device safety, and subgroup robustness associated with CGM use. The empirical cumulative distribution curves showed that patients in the CGM group tended to achieve ICU discharge earlier than those in the control group. Similarly, the cumulative distribution curve for mechanical ventilation duration suggested earlier liberation from mechanical ventilation among CGM users.

Subgroup analyses showed that the association between CGM use and lower in-hospital mortality was directionally consistent across prespecified subgroups, including age, sex, diabetes status, APACHE II tertiles, glucocorticoid use, and chronic kidney disease status. No significant interaction was observed across these subgroups, suggesting that the potential benefit of CGM-guided glucose management was generally robust across different clinical populations.

Sensitivity analyses with additional adjustment for age, sex, APACHE II score, chronic kidney disease, and glucocorticoid use yielded results that were generally consistent with the primary analyses. CGM use remained significantly associated with lower odds of in-hospital mortality (aOR, 0.49; 95% CI, 0.28–0.85; P = 0.011), severe hypoglycemia (aOR, 0.38; 95% CI, 0.20–0.74; P = 0.004), any hypoglycemia (aOR, 0.49; 95% CI, 0.32–0.73; P < 0.001), and nosocomial infection (aOR, 0.55; 95% CI, 0.33–0.90; P = 0.017). The estimate for ICU length of stay was unchanged from the primary analysis (aIRR, 0.86; 95% CI, 0.75–0.98; P = 0.021), whereas CGM use remained associated with a shorter duration of mechanical ventilation (aIRR, 0.71; 95% CI, 0.65–0.77; P < 0.001). In the fully adjusted Cox proportional hazards model, the association between CGM use and time to in-hospital death remained directionally consistent but did not reach statistical significance (aHR, 0.64; 95% CI, 0.40–1.01; P = 0.057; **Supplementary Table S1**).

Overall, CGM use was associated with more favorable glycemic, clinical, and ICU resource-utilization outcomes, although the time-to-event association with in-hospital mortality was less robust after additional adjustment.

## Discussion

In this single-center, retrospective, propensity score–matched cohort study, CGM use was associated with improved glycemic control, fewer hypoglycemic events, shorter ICU length of stay, and lower odds of in-hospital mortality. However, the time-to-event association with in-hospital mortality was attenuated and did not reach statistical significance after additional covariate adjustment.

Our findings are broadly consistent with previous clinical and observational studies evaluating CGM use in critically ill populations. Earlier randomized and observational studies have demonstrated that CGM enhances glucose stability and reduces hypoglycemia compared with conventional intermittent point-of-care testing. For instance, Holzinger et al. first showed that real-time CGM reduced the incidence of severe hypoglycemia in postoperative cardiac surgery patients without compromising overall glucose control (14). Evidence from an ICU randomized trial, critical care guidelines, and a review of CGM use in non-ICU hospital settings has described the potential role of CGM in supporting inpatient glucose management, while also emphasizing limitations in the available evidence (15–17). More recently, Shang et al. conducted a randomized controlled trial among frail and critically ill patients with COVID-19 and found that the use of intermittent-scanning isCGM significantly reduced 28-day ICU mortality, shortened ICU stay, and decreased both hypoglycemic and severe hypoglycemic events compared with standard point-of-care testing (18). Wang et al. performed a large multicentre observational study using isCGM to define hyperglycemia thresholds associated with mortality in critically ill adults. They identified 10.5 mmol/L as the critical inflection point beyond which time-above-range exposure was independently linked to increased in-hospital death, highlighting the prognostic value of CGM metrics (19).

Several mechanisms could plausibly explain an association between CGM-guided management and clinical outcomes, but none was tested as a mediator in this study (20, 21). More frequent trend information may facilitate earlier recognition of glucose excursions and may support insulin adjustment (22). Greater glucose stability has been associated with favorable outcomes in previous studies (23), although such associations do not establish that CGM itself causes improved survival. The accuracy and clinical implementation of CGM in hospitalized patients remain important considerations (24–26). Likewise, closed-loop or algorithm-guided insulin delivery was not evaluated and should not be inferred from these results.

Several limitations should be acknowledged. First, this retrospective observational study cannot establish causality, and propensity score matching cannot eliminate residual confounding. CGM allocation depended on physician judgment and device availability and may also have reflected glycemic instability, insulin use, clinician attention, nursing resources, nutrition, vasopressor exposure, and other unmeasured factors. Second, requiring an ICU stay of at least 72 hours excluded patients who died or were discharged earlier and may have introduced survivor-selection bias; the findings therefore apply primarily to patients remaining in the ICU for at least 72 hours. Third, CGM and POCT had markedly different measurement densities. Brief or asymptomatic glucose excursions could have been missed by POCT, and POCT proportions within or below range are not equivalent to CGM-derived TIR or TBR. Hypoglycemia duration could not be compared reliably. Fourth, standardized paired CGM–blood glucose measurements, edema severity, tissue-perfusion indices, and cumulative vasopressor doses were unavailable, precluding formal sensor-accuracy analyses such as MARD. Fifth, glucocorticoid dose and duration, daily carbohydrate delivery, feeding interruptions, and detailed insulin exposure were not consistently available. Finally, this was a single-center study, and long-term outcomes, cost-effectiveness, and nursing workload were not evaluated.

These real-world findings support further evaluation of CGM-guided glucose management in critically ill patients. They do not establish that CGM reduces mortality, infection, nursing workload, or other outcomes. Prospective multicenter studies using standardized glucose targets, insulin-adjustment algorithms, and measurement protocols should evaluate effectiveness, safety, cost-effectiveness, and workflow.

## Conclusion

In summary, CGM-guided glucose management was associated with improved glycemic stability and more favorable in-hospital outcomes among critically ill adults who remained in the ICU for at least 72 hours. Because the study was retrospective and subject to survivor-selection bias, residual confounding, and unequal glucose-measurement density, the findings should be interpreted as associations rather than evidence that CGM caused the observed clinical differences. Prospective multicenter studies are warranted.

## Data Availability

The datasets generated and analyzed during the current study are not publicly available because they contain potentially identifiable patient-level clinical information and are subject to institutional and ethical restrictions. Deidentified data may be made available from the corresponding author upon reasonable request, subject to approval by the Ethics Committee of The Affiliated Hospital of Qingdao University and applicable institutional data-sharing requirements.
