# Supplementary material for: Association of continuous glucose monitoring with in-hospital adverse outcomes among critically ill patients: a real-world propensity score–matched study
Source: Front Endocrinol (Lausanne). 2026 Aug 31;17:1921773. doi: 10.3389/fendo.2026.1921773 (PMC13572230; doi:10.3389/fendo.2026.1921773)
Supplement: Supplementary file 1 [file Table1.docx]

**Supplementary Table S1. Sensitivity analyses with additional adjustment for disease severity and clinically relevant covariates**

| **Outcome** | **CGM (n = 296)** | **Control (n = 296)** | **Unadjusted effect** | **Adjusted effect** **(sensitivity model)†** | **P value** |
| --- | --- | --- | --- | --- | --- |
| In-hospital mortality, n (%) | 28 (9.5) | 46 (15.5) | RR 0.61 (0.39–0.95) | aOR 0.49 (0.28–0.85) | 0.011 |
| Severe hypoglycemia <3.0 mmol/L, n (%) | 14 (4.7) | 32 (10.8) | RR 0.44 (0.24–0.80) | aOR 0.38 (0.20–0.74) | 0.004 |
| Any hypoglycemia <3.9 mmol/L, n (%) | 66 (22.3) | 102 (34.5) | RR 0.65 (0.50–0.84) | aOR 0.49 (0.32–0.73) | <0.001 |
| Nosocomial infection, n (%) | 36 (12.2) | 53 (17.9) | RR 0.68 (0.46–0.99) | aOR 0.55 (0.33–0.90) | 0.017 |
| ICU length of stay, days, median (IQR) | 7 (5–10) | 9 (6–13) | — | aIRR 0.86 (0.75–0.98) | 0.021 |
| Mechanical ventilation duration, days, median (IQR) | 4 (3–7) | 6 (4–9) | — | aIRR 0.71 (0.65–0.77) | <0.001 |
| Kaplan–Meier survival (CGM vs POCT) | — | — | Log-rank χ² = 4.1 | aHR 0.64 (0.40–1.01) | 0.057 |

Notes: IQR = interquartile range; RR = risk ratio; IRR = incidence rate ratio; aOR = adjusted odds ratio; aIRR = adjusted incidence rate ratio; aHR = adjusted hazard ratio; CI = confidence interval.

† Sensitivity model: binary outcomes were analyzed using multivariable GEE with a binomial distribution and logit link; ICU length of stay and mechanical ventilation duration were analyzed using negative binomial regression; and time-to-event survival was analyzed using a Cox proportional hazards model. All models were adjusted for age, sex, APACHE II score, chronic kidney disease, and glucocorticoid use. The matched-pair structure was accounted for using patient-pair clustering.
